# Wood Surface Finishing with Transparent Lacquers Intended for Indoor Use, and the Colour Resistance of These Surfaces during Accelerated Aging

**DOI:** 10.3390/polym15030747

**Published:** 2023-02-01

**Authors:** Jozef Kúdela, Adam Sikora, Lukáš Gondáš

**Affiliations:** 1 Department of Wood Science, Faculty of Wood Sciences and Technology, Technical University in Zvolen, T.G. Masaryka 24, 96001 Zvolen, Slovakia; 2 Department of Wood Processing and Biomaterials, Faculty of Forestry and Wood Sciences, Czech University of Life Sciences Prague, Kamýcká 129, 165 00 Prague, Czech Republic

**Keywords:** spruce wood, oak, surface treatment, solvent-based polyurethane lacquers, water-based lacquers, accelerated ageing, pigment mordants

## Abstract

This work evaluates the effects of accelerated aging on the discolouration of surface-treated spruce wood and oak wood coated with solvent-based polyurethane lacquers, and surface-treated spruce wood coated with water-based transparent coating systems. All concerned coating materials were intended for indoor use. It was also explored how the colour stability of spruce wood and oak wood surfaces treated with solvent-based polyurethane lacquers was affected by wood surface layer modifications with pigment or stain mordants applied before these lacquers. Another issue studied was how the lignin stabilizer admixed into the primer and pigments admixed into the top coating layers affected the stability of water-based coating systems on spruce. The experimental results showed that the accelerated aging process with a simulation of indoor conditions induced significant discolouration of wood surfaces coated with solvent-based polyurethane lacquers and water-based coating systems. There were also confirmed significant impacts of all the studied factors (wood species, lacquer/coating system type, lacquer modification, wood pre-treatment with pigment and stain mordants). The spruce wood surfaces coated with solvent-based polyurethane lacquers were less stable (Δ*E* = 10–19, dependent on the lacquer type) than the oak surfaces treated in the same ways (Δ*E* = 4–11). There were also confirmed significant impacts of the particular surface treatment on the colour stability as well as significant impacts of wood surface pre-treatment with pigment and stain mordants (Δ*E* = 4–17—for spruce wood, and Δ*E* = 5.5–13—for oak wood). In the case of water-based lacquers, the Δ*E* values ranged between 3 and 11 (according to the coating system type). The results show that an appropriate UV absorbent combined with an appropriate lignin stabilizer and pigment mordant may enable attaining the required colour stability for a given surface treatment applied on a given wood species.

## 1. Introduction

Surfaces of almost all materials exposed to outdoor conditions are affected by various types of radiation, moisture, heat, and pollutants. Acting in many interactions, these factors cause the surfaces to degrade progressively [1,2]. This problem is very relevant in the wood processing and furniture-making industry, as wood, being a natural, organic, and composite material, is from this viewpoint very specific compared to other materials. The products exposed to the environmental agents need their appropriate performance time guaranteed and the original physical and visual properties of their surface preserved. This is why the research in this area has long been oriented towards studying degradation, its causes, and possibilities of elimination, as well as studying a wide range of the factors possible to serve for affecting wood surface aging [3,4,5,6,7,8,9,10,11,12,13,14]. The results of a major part of the works related to the discussed issue have been summarised in [1,15]. A very common method of wood surface protection against such impacts is its surface treatment (ST) with coating materials (CM). CMs’ primary role is to significantly limit UV penetration into lignin and other wood components and to hinder, in this way, the degradation of surface wood layers. Additionally, ST needs to serve several other essential functions.

Moreover, the external environment negatively affects the ST quality as such [6,7,9,14,16,17,18,19,20,21,22,23,24,25,26,27,28]. The referred works show that the first degradation symptoms on wood surfaces treated with coating materials are present in surface treatment quality, primarily as changed visual properties such as colour and gloss, with a subsequent negative impact on the overall stability. The cited works also imply that the colour stability in surface-treated wood is species-specific and depends on the coating material transparency, type of organic or inorganic pigments, UV absorbers, lignin stabilizers, and similar factors. In the case of a colourless transparent surface treatment applied with the aim to pronounce the natural wood structure, the colour stability is significantly wood species-specific.

The problem is complex, demanding much effort and a multitask approach. Research is also needed in the context of today’s demands on ST durability and preserving the original quality. This is why the research has been oriented to improve ST colour stability, as well as overall stability, by means of targeted modification of the substrate and the coating material [14,16,17,18,19,21,29,30,31,32,33,34,35,36]. The referred works provide a considerable body of substantial knowledge related to the colour stability and the overall stability of surface-treated wood exposed to outdoor conditions. 

Distinctly, not as much interest has been given to the ageing process of sur-face-treated wood purposed for indoor conditions. The works [37,38,39] show that variations in indoor conditions primarily concern visual properties, especially discolouration. In comparison with outdoor conditions, the UV-induced effects in indoor conditions are less strong, but not negligible. Discolouration can also occur due to daylight comprising bigger wavelengths. Consequently, there has been an evident necessity for more attention regarding the effects of indoor conditions, especially related to the discolouration of surface-treated wood, and equally native wood without surface treatment [40,41]. The coating materials used for surface treatment of wood products intended for indoor performance need studying as a priority (furniture, indoor sides of window frames, and similar), with the aim that the application of these coating materials on wooden products can guarantee their long-lasting colour stability. Another important reason is that the surface treatment quality of indoor furniture pieces and of musical instruments is required to meet the most rigorous criteria.

The aim of this work was to evaluate the effects of the accelerated ageing process on the discolouration of surface-treated spruce wood and oak wood coated with solvent-based polyurethane lacquers, and of spruce wood coated with water-based coating systems intended for indoor use. Another objective was to analyse the impacts of modification of wood surface layers with pigment or stain mordants applied before the solvent-based polyurethane lacquers and to analyse in which way lignin stabiliser admixed to the primer and pigments admixed to the top layer may impact the colour stability of spruce wood coated with water-based lacquers.

## 2. Materials and Methods

### 2.1. Experimental Materials

Meeting the aims of the work required to lay out two experiments. The colour stability of four types of polyurethane lacquers was tested in the first experiment, labelled as PUR-1, PUR-2, PUR-3, and PUR-4 (PUR-1 is polyurethane topping lacquer based on polyester resin, PUR-2 is polyurethane lacquer with addition of acrylate and nitrocellulose, and PUR-3 and PUR-4 are polyurethane lacquers with addition of acrylate). The lacquers were two-component, solvent-based, transparent polyurethane lacquers intended for the surface finishing of furniture intended for indoor exposure, with improved mechanical and chemical resistance. The two last lacquers were identical, except that the lacquer PUR-4 was supplemented with a UV radiation adsorber acting on the benzotriazole base. In one case, the lacquers were applied directly on spruce and oak wood specimens sized 100 mm × 50 mm × 10 mm (length × width × thickness). In another case, the spruce wood surfaces were treated with a pigment mordant and oak wood specimens with a stain mordant, with the aim of inspecting mordant effects on the colour stability. The specimens stained in this way were finished with the tested lacquers. For each surface treatment type and for each wood species, there were prepared by 5 specimens, representing 80 in total. 

In the second experiment, four three-layer coating systems (CS) were tested for the surface treatment of wooden frames on the interior side of wood–aluminium windows. All the used coating systems were water-based. There was a mixture of acryl/alkyd or polyurethane resins and their combinations. All the coating materials were transparent. In this experiment, also studied was the influence of lignin stabiliser added to the primer, with the aim to slow down the colour degradation process. Another study aspect was to inspect in what way soft pigmentation would influence surface layer degradation. The pigments were admixed in amounts not suppressing the original spruce wood texture. The arrangement of CSs is in Table 1. In this experiment, the surface treatment was realized on surfaces of spruce specimens with the same dimensions as in the first experiment. Each particular coating system was used for the surface finishing of 6 specimens, representing 24 specimens. 

### 2.2. Accelerated Aging

The specimens with surfaces treated in this way were exposed to accelerated aging in a xenotest Q-SUN Xe-3-HS (Q-LabCorporation, Cleveland, OH, USA). The regimen simulated outdoor conditions when the material was exposed to radiation but protected from rain (Table 2). The indoor conditions were provided with Q-window filters simulating the light in the interior supplied by the daylight from the exterior. Table 1 illustrates the accelerated aging cycle consisting of two steps, with a total performance time of 120 min.

In accordance with the standard [42], the radiation intensity was set to 0.35 W·m^−2^ at a wavelength of *λ* = 340 nm. These values correspond to the average radiation intensity for the temperate zone. The temperature checked on a black panel reflects the maximum temperature value on the surface. The set air temperature accelerates the modification of surface properties. In the case of polyurethane lacquers, the colour changes were inspected after 50, 100, 200, 300, 400, and 500 h. On each specimen, the colour was measured at ten spots, so the total number of measurements for each surface ST mode was 50. In the case of water-based coating systems, the aging period was 700 h. 

The colour space coordinates L*, a*, and b* were measured on all specimens before and during the aging process. The measuring equipment was a spectrophotometer Spectro-Guide 45/0 gloss (BYK-GARDNER GmbH, Geretsried, Germany). The discolouration extent was expressed through the total colour difference Δ*E*, calculated according to the following equation.

(1) $$\Delta E=\sqrt{\Delta L^{2}+\Delta a^{2}+\Delta b^{2}}$$

where:

(2) $$\Delta L^{*}=L_{2}-L_{1},\;L_{3}-L_{1},...,\;L_{n}-L_{1}$$

(3) $$\Delta a^{*}=a_{2}-a_{1},a_{3}-a_{1},...,\;a_{n}-a_{1}$$

(4) $$\Delta b^{*}=b_{2}-b_{1},\;b_{3}-b_{1},...,b_{n}-b_{1}$$

Note tgat5 the indices “2−n” mean the colour values after wood surface irradiation, and the index “1” denotes the referential value measured on the wood surface before the aging process.

### 2.3. Statistical Evaluation

The results were processed in the programme STATISTICA version 10.0. The basic statistical characteristics (arithmetical mean, standard deviation) were determined with using the descriptive statistics. The impacts of the studied factors on the given properties were evaluated with multi-way variance analysis (MANOVA) and Duncan’s test.

## 3. Results and Discussion

### 3.1. Discolouration of Spruce Wood Surface Coated with Solvent-Based Polyurethane Lacquers

The average values of colour coordinates L*, a*, b*, and additional statistical characteristics measured during the aging process of spruce wood coated with polyurethane lacquers of four types are summarized in Table 3 and Table 4. The three-way variance analyses confirm that discoloration is significantly influenced by all three factors inspected (aging time, coating material type, modification with a UV absorbent). There were also confirmed significant effects of interaction between the relevant factors.

Table 3 shows that the values of colour coordinates L*, a*, and b* of spruce wood treated with polyurethane lacquers exhibited differences before aging. These differences were not much pronounced; nevertheless, they reflected the fact that the tested transparent lacquers were not absolutely pure. This was especially evident for the lacquers PUR-1 and PUR-2. In these cases, the coordinate b* value was shifted toward more saturated yellow, and the a* value was shifted toward red, in comparison to the native wood surface. In the presence of a slight lightness reduction, the spruce wood exhibited a darker yellow-brown hue after surface finishing. The values of the total colour difference Δ*E* were within a range between 1 and 2.0, which corresponds to the second degree of the six-degree scale [43]. These differences, however, need not be visible instantly due to the variability in the spruce wood texture and colour, issuing mainly from the differences between the early and late wood colouring.

**Figure 1:**
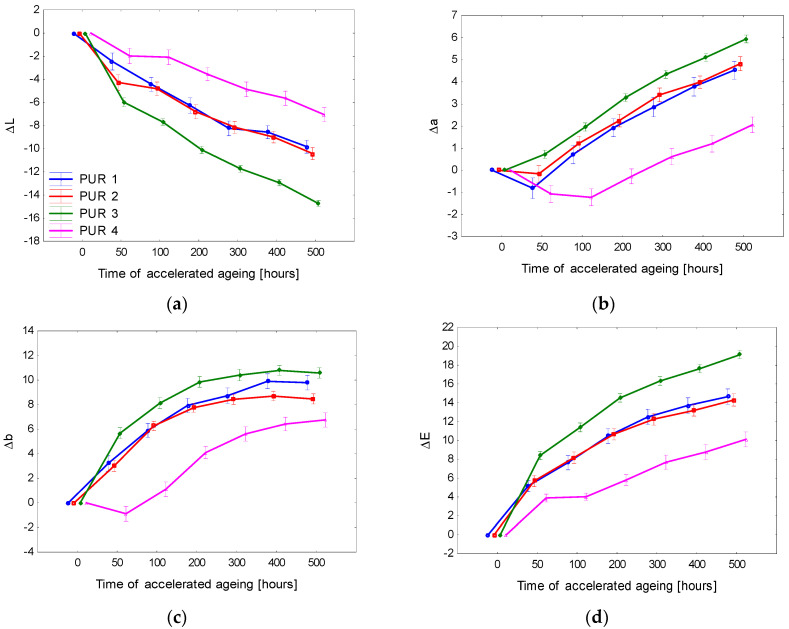
Accelerated-aging-induced differences in colour coordinates (**a**) ΔL*, (**b**) Δa*, and (**c**) Δb*, and the total colour difference (**d**) ΔE* of spruce wood surfaces finished with polyurethane lacquers on surfaces without pigment mordants.

**Figure 2:**
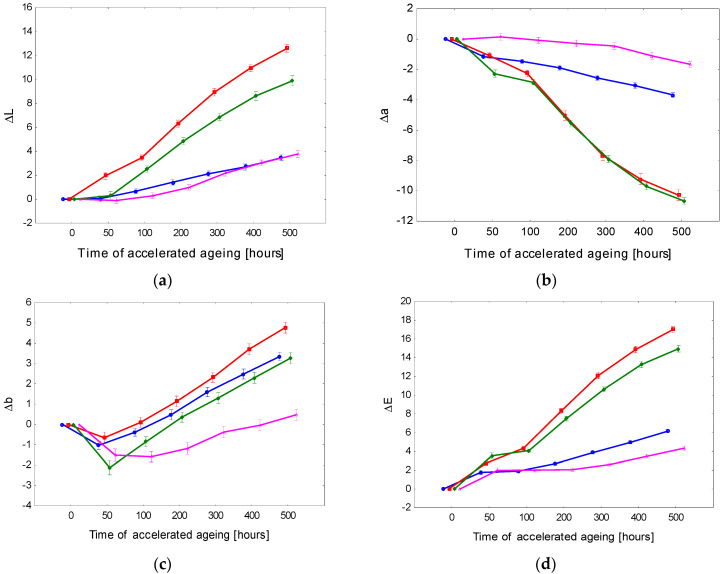
Accelerated-aging-induced differences in colour coordinates (**a**) ΔL*, (**b**) Δa*, and (**c**) Δb*, and the total colour difference (**d**) ΔE* of spruce wood surfaces finished with polyurethane lacquers on surfaces with pigment mordants.

The lightness of surface-treated spruce wood decreased with advancing aging time in all cases (Figure 1 and Figure 2). After 500 accelerated aging hours, the most conspicuous lightness variation was observed in the case of the lacquer PUR-3 (decrease by a value of 15). In the case of PUR-1 and PUR-2, the lightness reduction represented 10. All three lacquers were without stabilizers, and they differed in their chemical structure. The final lightness of the spruce wood surface finished with these lacquers was practically the same (L* ≅ 63.5). The finishing with the lacquer PUR-4 exhibited the minimum lightness modification. In this case, the effect of the UV absorber added was evident.

**Figure 3:**
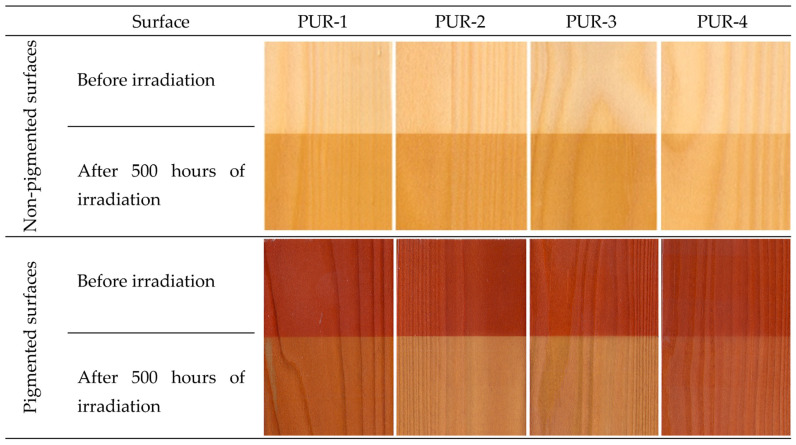
Colour of spruce wood surfaces coated with specific lacquers before and after irradiation in the accelerated aging process.

The values of coordinate a* increased with aging time up to the end of the aging process. This means that the coordinate a* was growing moderately towards red saturation. A qualitatively similar course was also recorded for the coordinate b*, with the values step-by-step shifting to yellow. As a result, the surfaces treated with the tested polyurethane lacquers darkened progressively, turning to a darker yellow-brown hue compared to the referential specimens (Figure 3).

The changes in the colour coordinates ΔL*, Δa*, and Δb* were also reflected in the total colour difference Δ*E* (Figure 1 and Figure 2). The total colour differences for the particular surface finishing types were evaluated based on a six-degree scale proposed by [43]. The evaluation resulted in finding degree 5, identifying noticeable colour change in the surface treatment PUR-3 as early as after 50 aging hours. After 200 h, the colour change corresponded to degree 6, expressing a new colour compared to the original. The change in the ST with the lacquers PUR-1 and PUR-2 was similar, only less rapid. Furthermore, in the case of these two lacquers, the final colour difference after 500 aging hours corresponded to degree 6.

Spruce wood surfaces demonstrated the poorest colour stability finished with a lacquer PUR-3. Supplementing this lacquer with a solvent-based UV absorber (obtained PUR-4) significantly improved the colour stability of the surface treatment. After 200 aging hours, the overall colour differences in the spruce wood surface finished with PUR-4 corresponded to degree 4 (change visible through a medium-quality filter). After the aging process had finished, the discolouration corresponded to degree 5. The protective substance applied in this coating system significantly decelerated photodegradation. Nevertheless, it was not effective enough to suppress the colour changes on the spruce wood surface to negligible changes.

The data concerning the colour variation on the surface-treated spruce wood preliminary modified with pigment stain mordents are presented in Table 4 and in Figure 2 and Figure 3. Furthermore, in this case, the results of the three-way variance analysis revealed that all three discussed factors and their interactions significantly affected the variability of the colour space CIE L*a*b*.

The pre-treatment of the spruce wood surface with a pigment stain mordant commonly used for attaining the required colour hue of the wood surface also enabled guaranteeing of better colour homogeneity. In this case, the changes in the colour coordinates differed from the corresponding changes obtained for non-stained surfaces, both in quality and quantity (Figure 2 and Figure 3). In almost all cases (except PUR-1), the lightness was enhanced with prolonged aging time. This change, however, was not as distinct as in the former case. The values of the coordinate a* decreased moderately. Contrarily, the values of coordinate b* rose and shifted towards yellow. Figure 2 shows the most conspicuous changes in the lacquers PUR-2 and PUR-3. In these lacquers, the total colour change after finishing the aging process reached the highest degree of the six-degree scale. The discolouration of the wood surface finished with PUR-1 and PUR-4 did not reach beyond 4. The colour stability of surface-treated spruce wood was considerably improved by preliminary treatment with a pigment mordant. Nevertheless, the changes associated with the accelerated aging process remained significant.

### 3.2. Discolouration of Oak Wood Surface Coated with Solvent-Based Polyurethane Lacquers

**Figure 4:**
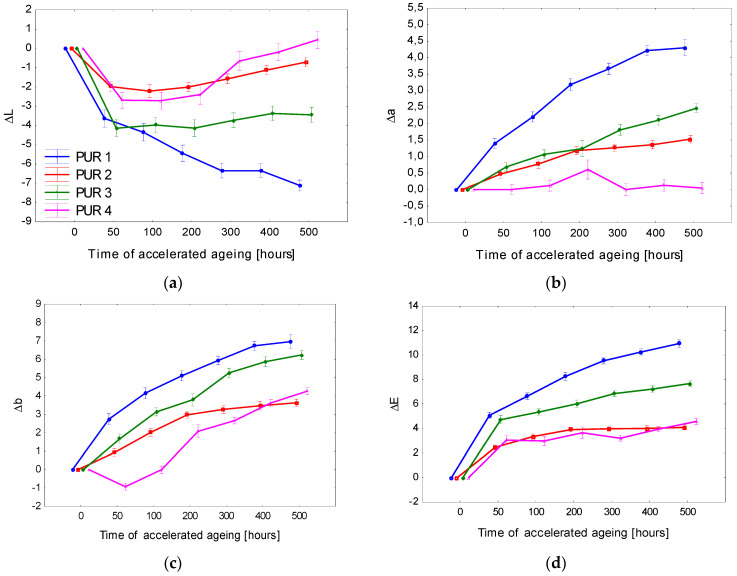
Accelerated-aging-induced differences in colour coordinates (**a**) ΔL*, (**b**) Δa*, and (**c**) Δb*, and the total colour difference (**d**) ΔE* of oak wood surfaces finished with polyurethane lacquers on surfaces without pigment mordants.

**Figure 5:**
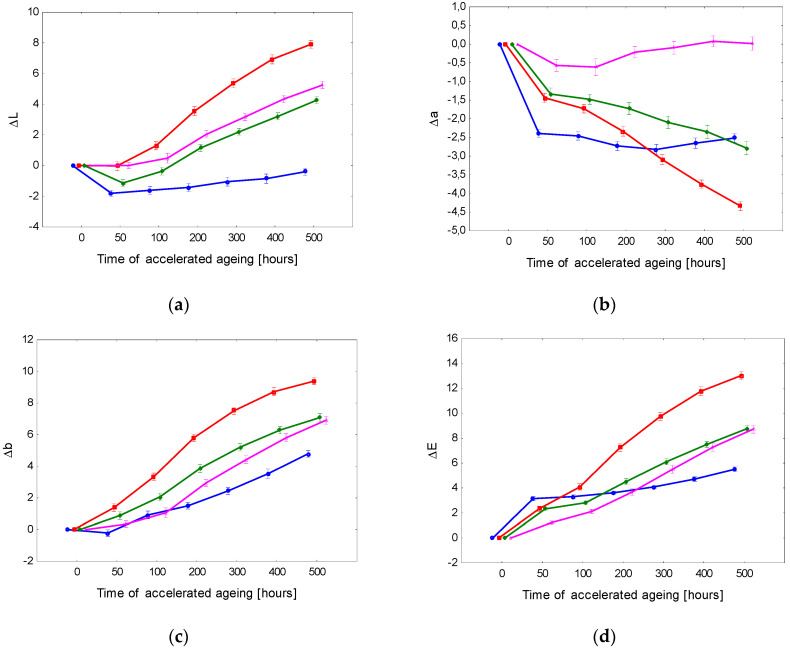
Accelerated-aging-induced differences in colour coordinates (**a**) ΔL*, (**b**) Δa*, and (**c**) Δb*, and the total colour difference (**d**) ΔE* of oak wood surface finished with polyurethane lacquers on surfaces with pigment mordants.

The same lacquers were also applied onto oak wood. The results are summarized in Table 5 and Table 6. Additionally, in this case, the colour variations during the accelerated aging process were backed-up by all the studied factors. Significant impacts on colour stability were found for interactions between these factors. The differences in the colour coordinates as well as the total colour difference, are shown in Figure 4 and Figure 5.

**Figure 6:**
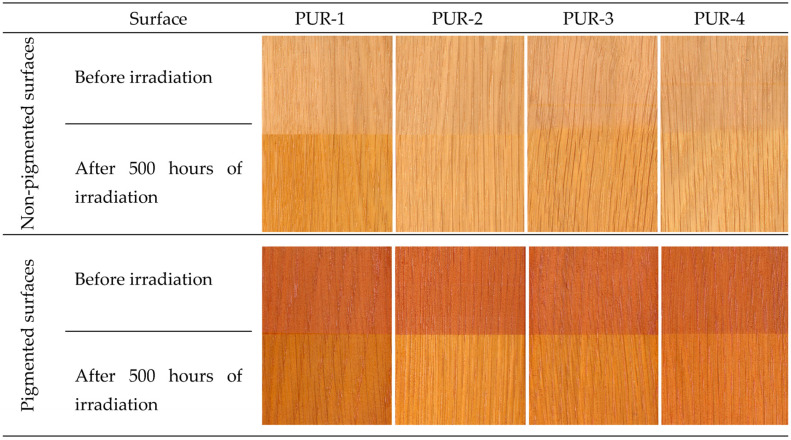
Colour of oak wood surfaces coated with specific lacquers before and after irradiation in the accelerated aging process.

The results showed that the colour stability of surface-treated oak wood was different from spruce in extent and patterns (Table 5). In general, we can state that the overall discolouration after finishing the aging process was less evident than in the case of spruce wood. In the case of PUR-1, the lightness decreased during the aging process; in the other cases, the decrease occurred during the initial 50 h, and a moderate increase followed. In the case of PUR-4 surface treatment, the final change in lightness was practically zero. As a consequence of a moderate increase in a* and especially the b* coordinate during aging, the surfaces exhibited more saturated yellow-brown hues (Figure 6). Similarly, the best colour stability was observed in oak wood surfaces coated with PUR-4, followed by PUR-2, and the least stable were PUR-1-finished surfaces. We can see that the colour-stability-based ranking was different from spruce. 

In no case did the total colour difference value Δ*E* reach beyond 12. The discolouration of the surfaces finished with PUR-2 and PUR-4 was between degrees 3 and 4, in the case of PUR-1 and PUR-3, it was degree 5 of the six-degree scale. Better colour stability is supposedly due to the different chemical structures of oak wood, especially extractives causing the oak wood to be darker than spruce. The works [7,9,23,33,37] stated that the response to UV radiation is more sensitive in the natively light-coloured wood species.

The discolouration of surface-treated oak wood preliminarily coated with a stain mordant exhibited qualitatively different discolouration compared to the unstained wood (Table 6 and Figure 5 and Figure 6). In contrast, the lightness of these surfaces increased with aging. The colour coordinate a* values moderately decreased (except PUR-4), while the coordinate b* values increased. In this case, the highest colour stability was observed for the surfaces treated with PUR-1, followed by PUR-4 coated surfaces, and finishing with PUR-2. The results indicate more conspicuous discolouration in the stained oak wood compared to unstained (see Figure 6). Evidently, the result is opposite to the case of spruce wood and also beech wood stained with a mordant [37].

### 3.3. Discolouration of Oak Wood Surface Coated with Solvent-Based Polyurethane Lacquers

**Figure 7:**
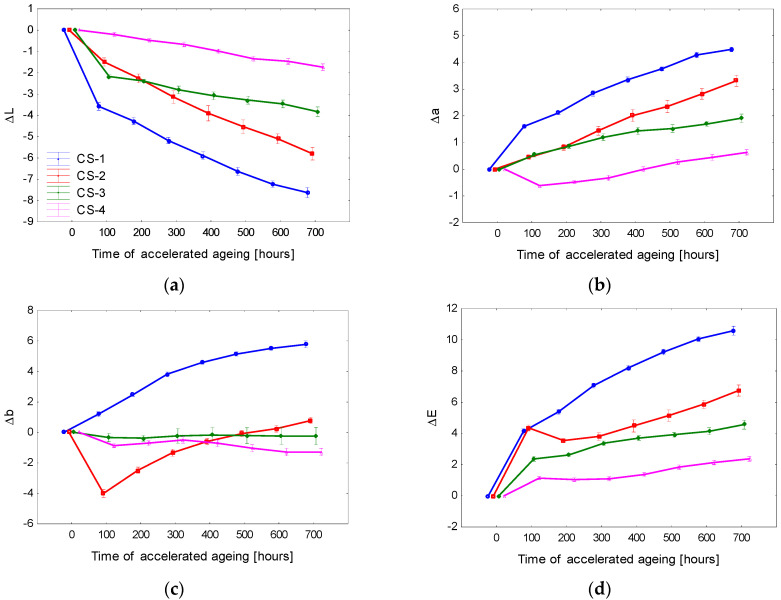
Accelerated-aging-induced differences in colour coordinates (**a**) ΔL*, **(b**) Δa*, and (**c**) Δb*, and the total colour difference (**d**) ΔE* in spruce wood surfaces coated with water-based coating systems.

The basic statistical characteristics of the colour coordinates corresponding to the particular accelerated ageing phases are in Table 7. The differences in the colour coordinates ΔL*, Δa*, and Δb* and the total colour difference Δ*E* for the tested coating systems applied onto spruce wood are displayed in Figure 7. The results summarised in Table 7 and in Figure 7 show that the average lightness value L* in specimens with surfaces treated with transparent three-layer coating systems (CS-1 and CS-2) was 82. After admixing the white pigment (CS-3, CS-4), the average lightness value rose to 86. With advancing irradiation time, in all cases, these lightness values moderately decreased until the aging process was finished.

Before the aging, the coordinate a* values in the particular surface treatment types ranged between 2 and 4. With progressing aging, the coordinate a* values increased in all cases, which means that this coordinate shifted towards a more saturated red colour (Figure 7). Figure 7 demonstrates that coordinate b* exhibited the smallest changes related to the accelerated aging process. 

The shifts in all three coordinates L*, a*, and b* exhibited significant differences between the individual coating systems. These changes were also reflected in the total colour difference Δ*E* (Figure 7). The most obvious variation in the colour coordinates and also in the total colour difference was observed on the spruce wood surface treated with the system CS-1. All three lacquer layers were transparent and colourless, without any modifications. After 700 aging hours, the value of Δ*E* reached close to 12, corresponding, according to the six-degree scale [43], to the degree five or six, expressing notable discolouration or a new colour evaluated as different from the original colour.

**Figure 8:**
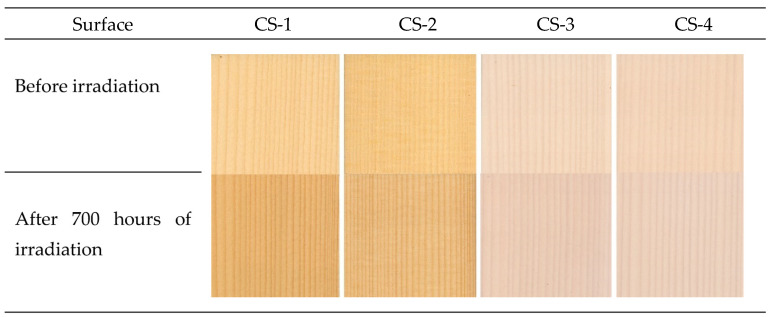
Colour of spruce wood surfaces coated with specific water-based coating systems before and after irradiation in the accelerated aging process.

The structure of the coating system CS-2 was the same as CS-1, with the difference that the primer was supplemented with a lignin stabilizer. This modification of the primer resulted in significantly improved stability of the spruce wood surface treatment (Figure 7 and Figure 8). After finishing the aging process in indoor and outdoor conditions, the total colour difference still surpassed 6, expressing a noticeable discoloration according to the mentioned scale. Coating systems CS-3 and CS-4 exhibited, thanks to the pigment admixed, more colour stability than CS-1 and CS-2. The coating system CS-3 was less steady than CS-4 because, in the case of CS-3, the pigment was admixed into the top layer only, and in the case of CS-4, in addition, lignin stabilizer was added into the primer. It follows that the surface treatment CS-4 with the top glazing layer was found as the most proper. In this coating system, Δ*E*, after 700 aging hours, remained below degree 2, which indicates only slight discolouration.

The authors of [30] report that gloss loss in transparent coatings indicates degradation of the coating alone, while their discolouration may be due to the photodegradation of the coating and the substrate equally. The differences in photodegradation between spruce, oak, and beech wood [37] with surfaces coated with identical lacquers reflect mainly substrate-related differences in photodegradation. Better colour resistance of surface-treated oak wood compared to spruce and even to beech [37] confirms the already-recognised fact that darker wood species are more stable. This has also been confirmed with the results of the work [39]. However, the wood species, pigment, and stain type were also important factors. The literature references report several other factors potentially improving wood surface treatment stability: heat pre-treatment, nanoparticles, plasma treatment, and similar [39,41,44,45]. Work [33] providing the testing results for nine colour hues confirmed that the pigments exerted considerable inhibiting effects on photodegradation, with darker pigment hues showing more efficiency. Similar effects of pigmentation are described in [31,37,46]. Our results, and equally the results of the last cited works, imply that it is necessary to distinguish between coating substance modification with pigments and wood surface modification with pigmented stains [33,46]. The results also imply that an appropriate combination of a UV absorber, a lignin stabilizer, and a mordant can enable colour stability for surface treatment systems applied on the relevant wood species.

## 4. Conclusions

Experimental results showed that an accelerated aging process with the simulation of indoor conditions induced significant discolouration of surface-treated wood, both coated with solvent-based polyurethane as well as water-based lacquers. There were also confirmed significant impacts for all the studied parameters (wood species, lacquer/coating system type, lacquer modification, and wood surface pre-treatment with pigment and stain mordants).

The lightness of surface-treated spruce wood and oak wood coated with polyurethane lacquers decreased with aging. The values of coordinates a*, b* moderately increased with aging time. As a result, the surfaces coated with the tested polyurethane lacquers became darker, shifting to darker yellow-brownish hues compared to the referential specimens. In general, we may declare that, after finishing the aging process, the discolouration of the surface-treated oak wood was less distinct compared to the spruce wood. 

From the viewpoint of colour stability, the ranking of spruce wood surfaces coated with the discussed lacquers was different from oak. In both cases, the best colour stability was found for the lacquer PUR-4 supplemented with UV absorbent. The poorest colour stability in the surface-treated spruce wood was observed for the lacquer PUR-3, and for the oak wood, it was the lacquer PUR-1.

The variation in colour coordinates in surface-treated spruce wood and oak wood coated with identical lacquers showed qualitative and quantitative differences between the surfaces pre-treated with pigment or stain mordants and the surfaces without this pre-treatment. From this viewpoint, there were also observed differences in the ranking according to the surface colour stability.

Pre-treatment of spruce wood surfaces with pigment/stain mordants improved the overall colour stability. Nevertheless, the accelerated aging-induced discolouration remained significant. In the case of oak wood, no positive influence of mordant treatment on colour stability was detected. 

Similarly, spruce wood surfaces coated with water-based coating systems exhibited distinct discolouration associated with the accelerated aging process. There were noticeable differences between the studied coating systems in all three colour coordinates and in the resulting total colour difference Δ*E*. The most conspicuous change was observed in the spruce wood coated with the system CS-1, with all three layers transparent and colourless, without any modification. Lignin stabilizer admixed to the primer and white pigment admixed to the top layer significantly improved the colour stability of the surface-treated spruce wood (CS-4).

The analysis of the results allows drawing of the conclusion that the colour variations in the studied surface-treated wood species subject to the accelerated aging process are mainly caused by colour degradation of the surface wood layers on their own.

## Figures and Tables

**Table 1:** Structure of coating systems applied on spruce wood.

CS	SCS Structure	Explanations
1	P1 + ML + F1	P1—colourless primer without lignin stabilizer P2—colourless primer with lignin stabilizer ML—colourless middle layer F1—upper finishing layer colourless F2—upper finishing layer, soft pigmented with a white pigment
2	P2 + ML + F1	
3	P1 + ML + F1	
4	P2 + ML + F2	

**Table 2:** The aging parameters set according to the Standard ASTM G 155 [42].

Step	Mode	Radiation Intensity (W/m²)	Black Panel Temperature (°C)	Air Temperature (°C)	Relative Air Humidity (%)	Time (min.)
1	Radiation	0.35	63	48	30	102
2	Radiation-free	-	-	38	–	18

**Table 3:** Basic statistical characteristics of colour coordinates L^*^, a^*^, and b^*^ corresponding to different phases of the accelerated aging process of spruce wood surface-treated with polyurethane lacquers. (The number of measurements for each set n = 50.)

Lacquer Type	Colour Coordinates	Basic Statistical Characteristics	Aging Time (hours)						
			0	50	100	200	300	400	500
PUR-1	L*	$\bar{x}$	73.66	71.21	69.24	67.40	65.42	65.09	63.83
		*s*	1.96	1.86	1.06	1.30	1.50	0.85	0.91
	a*	$\bar{x}$	8.45	7.63	9.16	10.37	11.30	12.23	12.97
		*s*	1.44	0.98	0.75	0.70	0.78	0.58	0.57
	b*	$\bar{x}$	29.21	32.42	35.11	37.15	37.94	39.11	39.00
		*s*	2.07	0.90	0.65	0.59	1.16	0.50	0.54
PUR-2	L*	$\bar{x}$	73.81	69.54	68.99	67.01	65.67	64.82	63.39
		*s*	1.62	1.82	1.05	1.07	0.70	0.64	0.61
	a*	$\bar{x}$	8.28	8.13	9.51	10.54	11.70	12.28	13.11
		*s*	0.95	0.91	0.53	0.41	0.45	0.30	0.41
	b*	$\bar{x}$	30.45	33.47	36.73	38.21	38.89	39.15	38.92
		*s*	1.25	0.79	0.50	0.53	0.49	0.48	0.45
PUR-3	L*	$\bar{x}$	78.32	72.35	70.64	68.22	66.59	65.38	63.60
		*s*	0.82	1.05	0.46	0.62	0.49	0.29	0.29
	a*	$\bar{x}$	6.23	6.96	8.20	9.53	10.58	11.35	12.18
		*s*	0.61	0.28	0.21	0.25	0.26	0.14	0.14
	b*	$\bar{x}$	27.10	32.78	35.22	36.95	37.51	37.89	37.68
		*s*	1.49	0.61	0.55	0.47	0.63	0.30	0.33
PUR-4	L*	$\bar{x}$	76.49	74.52	74.41	72.94	71.63	70.86	69.45
		*s*	2.08	1.49	1.15	0.75	0.87	0.59	0.78
	a*	$\bar{x}$	7.08	6.02	5.86	6.81	7.70	8.29	9.14
		*s*	1.35	0.83	0.66	0.58	0.62	0.51	0.58
	b*	$\bar{x}$	28.41	27.53	29.53	32.50	34.03	34.84	35.17
		*s*	1.88	1.44	1.05	1.09	1.23	0.97	1.61

**Table 4:** Basic statistical characteristics of colour coordinates L^*^, a^*^, and b^*^ corresponding to different phases of accelerated aging of spruce wood surface-treated polyurethane lacquers—the wood surface was pre-treated with a pigment stain. (The number of measurements in each set n = 50.)

Lacquer Type	Colour Coordinates	Basic Statistical Characteristics	Aging Time (hours)						
			0	50	100	200	300	400	500
PUR-1	L*	$\bar{x}$	40.44	40.48	41.11	41.86	42.56	43.13	43.90
		*s*	0.73	0.64	0.62	0.56	0.61	0.59	0.63
	a*	$\bar{x}$	24.12	22.96	22.66	22.23	21.57	21.06	20.44
		*s*	0.53	0.40	0.38	0.24	0.36	0.45	0.25
	b*	$\bar{x}$	19.42	18.40	19.04	19.90	21.02	21.90	22.77
		*s*	0.77	0.55	0.71	0.61	0.91	0.78	0.90
PUR-2	L*	$\bar{x}$	39.90	41.86	43.36	46.22	48.83	50.86	52.49
		*s*	1.11	0.69	0.80	1.09	1.18	1.22	1.04
	a*	$\bar{x}$	24.16	23.07	21.90	19.13	16.49	14.93	13.87
		*s*	0.70	0.36	0.27	0.58	0.70	0.77	0.73
	b*	$\bar{x}$	21.22	20.56	21.32	22.35	23.53	24.92	25.99
		*s*	0.93	0.53	0.48	0.49	0.48	0.49	0.45
PUR-3	L*	$\bar{x}$	40.08	40.37	42.59	44.92	46.94	48.70	49.96
		*s*	0.89	0.89	0.61	0.64	0.65	0.85	1.02
	a*	$\bar{x}$	25.21	22.92	22.37	19.65	17.31	15.50	14.55
		*s*	0.60	0.67	0.28	0.59	0.63	0.65	0.56
	b*	$\bar{x}$	22.54	20.42	21.68	22.90	23.85	24.84	25.81
		*s*	0.93	0.80	0.44	0.41	0.27	0.42	0.38
PUR-4	L*	$\bar{x}$	39.82	39.71	40.10	40.79	41.98	42.84	43.59
		*s*	0.86	0.81	0.69	0.75	0.80	0.80	0.97
	a*	$\bar{x}$	25.20	25.35	25.13	24.92	24.75	24.10	23.55
		*s*	0.58	0.50	0.36	0.54	0.37	0.44	0.37
	b*	$\bar{x}$	22.44	20.93	20.85	21.26	22.07	22.40	22.92
		*s*	0.85	0.73	0.56	0.67	0.52	0.41	0.59

**Table 5:** Basic statistical characteristics of colour coordinates L^*^, a^*^, and b^*^ corresponding to different phases of accelerated aging of oak wood surfaces treated with solvent-based polyurethane lacquers. (The number of measurements in each set: n = 50.)

Lacquer Type	Colour Coordinates	Basic Statistical Characteristics	Aging Time (hours)						
			0	50	100	200	300	400	500
PUR-1	L*	$\bar{x}$	63.11	59.48	58.75	57.67	56.77	56.77	56.00
		*s*	1.45	0.81	0.70	0.77	0.65	0.61	1.16
	a*	$\bar{x}$	9.56	10.96	11.76	12.74	13.22	13.77	13.86
		*s*	0.36	0.37	0.40	0.51	0.48	0.44	0.71
	b*	$\bar{x}$	26.97	29.71	31.12	32.07	32.91	33.70	33.93
		*s*	0.78	0.45	0.58	0.47	0.60	0.63	1.28
PUR-2	L*	$\bar{x}$	63.20	61.22	60.98	61.20	61.62	62.08	62.49
		*s*	1.01	0.76	1.02	0.85	0.72	0.67	0.57
	a*	$\bar{x}$	8.98	9.45	9.75	10.16	10.24	10.34	10.51
		*s*	0.32	0.52	0.45	0.48	0.30	0.39	0.36
	b*	$\bar{x}$	28.49	29.41	30.51	31.47	31.76	31.96	32.12
		*s*	0.54	0.41	0.50	0.43	0.43	0.58	0.45
PUR-3	L*	$\bar{x}$	62.86	58.72	58.89	58.73	59.13	59.49	59.41
		*s*	1.34	1.09	0.80	1.09	0.88	0.84	0.78
	a*	$\bar{x}$	9.87	10.56	10.94	11.11	11.68	11.97	12.34
		*s*	0.28	0.56	0.55	0.87	0.62	0.59	0.52
	b*	$\bar{x}$	27.66	29.32	30.79	31.44	32.91	33.52	33.89
		*s*	0.61	0.63	0.49	1.36	0.65	0.69	0.60
PUR-4	L*	$\bar{x}$	62.25	59.56	59.54	59.86	61.60	62.07	62.70
		*s*	1.72	1.72	1.84	1.52	1.63	1.63	1.64
	a*	$\bar{x}$	9.79	9.79	9.91	10.40	9.79	9.92	9.84
		*s*	0.50	0.46	0.52	0.72	0.51	0.52	0.38
	b*	$\bar{x}$	28.10	27.17	28.10	30.17	30.75	31.72	32.36
		*s*	0.56	0.44	0.51	1.24	0.51	0.48	0.60

**Table 6:** Basic statistical characteristics of colour coordinates L^*^, a^*^, and b^*^ corresponding to different phases of accelerated aging of oak wood surfaces treated with solvent-based polyurethane lacquers. (The number of measurements in each set: n = 50.)

Lacquer Type	Colour Coordinates	Basic Statistical Characteristics	Aging Time (hours)						
			0	50	100	200	300	400	500
PUR-1	L*	$\bar{x}$	49.75	47.95	48.14	48.33	48.71	48.90	49.35
		*s*	0.61	0.54	0.66	0.71	0.73	0.87	0.62
	a*	$\bar{x}$	20.24	17.84	17.78	17.51	17.41	17.58	17.72
		*s*	0.30	0.27	0.25	0.27	0.30	0.32	0.23
	b*	$\bar{x}$	25.69	25.48	26.61	27.19	28.13	29.22	30.47
		*s*	0.59	0.42	0.65	0.62	0.58	0.87	0.68
PUR-2	L*	$\bar{x}$	49.14	49.12	50.43	52.70	54.50	56.06	57.05
		*s*	1.03	0.68	0.63	0.71	0.69	0.62	0.73
	a*	$\bar{x}$	21.73	20.29	20.00	19.39	18.64	17.98	17.39
		*s*	0.37	0.32	0.32	0.37	0.32	0.22	0.26
	b*	$\bar{x}$	27.50	28.88	30.85	33.31	35.01	36.22	36.88
		*s*	0.65	0.58	0.55	0.64	0.65	0.67	0.63
PUR-3	L*	$\bar{x}$	49.41	48.27	49.04	50.56	51.61	52.62	53.67
		*s*	0.73	0.49	0.61	0.52	0.45	0.49	0.54
	a*	$\bar{x}$	21.59	20.25	20.11	19.86	19.49	19.24	18.80
		*s*	0.56	0.32	0.35	0.29	0.33	0.33	0.31
	b*	$\bar{x}$	28.02	28.90	30.09	31.88	33.23	34.30	35.10
		*s*	0.76	0.30	0.41	0.38	0.43	0.48	0.49
PUR-4	L*	$\bar{x}$	46.62	46.63	47.11	48.66	49.79	50.95	51.87
		*s*	1.03	0.57	0.46	0.74	0.80	0.78	0.64
	a*	$\bar{x}$	21.25	20.68	20.64	21.04	21.16	21.33	21.27
		*s*	0.53	0.28	0.44	0.32	0.38	0.27	0.28
	b*	$\bar{x}$	26.30	26.65	27.36	29.24	30.72	32.10	33.22
		*s*	0.97	0.43	0.37	0.55	0.58	0.63	0.53

**Table 7:** Basic statistical characteristics of colour coordinates L^*^, a^*^, and b^*^ correspond to different phases of accelerated aging of oak wood surfaces treated with water-based coating systems. (The number of measurements in each set: n = 50.)

Coating System	Colour Coordinates	Basic Statistical Characteristics	Aging Time (hours)							
			0	100	200	300	400	500	600	700
CS-1	L*	$\bar{x}$	82.67	79.09	78.40	77.49	76.77	76.04	75.44	75.04
		*s*	0.28	0.63	0.60	0.61	0.62	0.66	0.53	0.42
	a*	$\bar{x}$	2.80	4.40	4.91	5.64	6.15	6.56	7.07	7.29
		*s*	0.16	0.14	0.12	0.20	0.19	0.14	0.22	0.30
	b*	$\bar{x}$	26.39	27.59	28.87	30.23	30.99	31.54	31.91	32.17
		*s*	0.27	0.46	0.26	0.30	0.33	0.46	0.43	0.45
CS-2	L*	$\bar{x}$	81.54	80.05	79.29	78.40	77.64	77.02	76.44	75.74
		*s*	0.34	0.30	0.33	0.54	0.70	0.93	0.72	0.70
	a*	$\bar{x}$	3.66	4.11	4.49	5.11	5.67	6.02	6.49	6.98
		*s*	0.12	0.17	0.26	0.43	0.56	0.66	0.57	0.53
	b*	$\bar{x}$	28.69	24.70	26.22	27.37	28.10	28.61	28.94	29.48
		*s*	0.48	0.51	0.31	0.32	0.22	0.28	0.27	0.18
CS-3	L*	$\bar{x}$	85.72	83.54	83.33	82.91	82.64	82.44	82.25	81.89
		*s*	0.79	0.71	0.80	0.94	0.99	0.76	0.97	1.25
	a*	$\bar{x}$	2.23	2.77	3.08	3.43	3.66	3.75	3.92	4.14
		*s*	0.33	0.29	0.31	0.41	0.46	0.30	0.40	0.65
	b*	$\bar{x}$	13.57	13.24	13.20	13.35	13.40	13.37	13.34	13.34
		*s*	2.77	2.12	2.49	2.76	2.84	2.97	3.04	3.12
CS-4	L*	$\bar{x}$	85.60	85.39	85.13	84.93	84.61	84.26	84.14	83.86
		*s*	0.12	0.20	0.19	0.25	0.22	0.24	0.34	0.39
	a*	$\bar{x}$	2.54	1.93	2.05	2.21	2.53	2.81	2.98	3.17
		*s*	0.09	0.12	0.19	0.26	0.23	0.22	0.26	0.25
	b*	$\bar{x}$	13.72	12.86	13.02	13.23	13.01	12.69	12.43	12.44
		*s*	0.49	0.47	0.36	0.25	0.30	0.36	0.46	0.47

## Data Availability

The data that support the findings of this study are available on request from the corresponding author.
